# Correlation of Diabetic Status and Coronavirus Disease 2019 (COVID-19) in Patients With Mucormycosis: A Retrospective Clinical Study

**DOI:** 10.7759/cureus.48936

**Published:** 2023-11-17

**Authors:** Madhumitha M, Devika S Pillai

**Affiliations:** 1 Department of Oral Medicine and Radiology, Saveetha Dental College and Hospitals, Saveetha Institute of Medical and Technical Sciences (Deemed to be University), Chennai, IND

**Keywords:** covid-19, radiographic features, clinical spectrum, diabetes, mucormycosis

## Abstract

Aim: Coronavirus disease 2019 (COVID-19) and its association with diabetes might lead to mucormycosis, and studies have reported an association between them. This study aims to find the correlation between COVID-19 and diabetic status in patients with mucormycosis and its role in disease progression and prognosis. The objectives of the study are to analyze the clinical range of mucormycosis in those with diabetes and COVID-19 and to correlate the clinical and radiographic findings.

Materials and methodology: A retrospective cohort analysis was carried out at Saveetha Dental College and Hospitals in Chennai (approval number: IHEC/SDC/OMED-2204/23/218). The data collection was done from the institution's electronic database from April 2019 to April 2023 which included the patients' age and gender and COVID-19 and diabetic status and clinical and radiographic features of mucormycosis.

Results: From the data analyzed, 25 patients had a history of mucormycosis with diabetes and COVID-19 infections. The patients' average age was 47.76, out of which 22 were males and three were females. The chi-squared test showed no significant association between age (0.178), diabetes (0.465), and COVID-19 (0.583). Spearman's correlation was done showing an association between mucormycosis, diabetes, and COVID-19. Radiographically, 100% of the patients had involvement of the maxillary sinus, followed by the palate (32%), orbit (28%), nasal floor (24%), ethmoidal sinus (16%), sphenoidal sinus (12%), and frontal sinus (8%).

Conclusion: The findings of this study point out the importance of considering the presence of systemic comorbidities like diabetes in COVID-19 patients. Early identification, surgical debridement, and antifungal medications are part of the treatment for increased survival.

## Introduction

The coronavirus disease 2019 (COVID-19) pandemic has been brought on by the severe acute respiratory syndrome coronavirus 2 (SARS-CoV-2) virus [1]. The coronavirus is a member of the family *Coronaviridae* and the order *Nidovirales*. Its viral surface has spikes that resemble crowns. Consequently, it has been given the name coronavirus. As the coronavirus has confirmed its presence worldwide, it is well-known everywhere. The International Committee on Taxonomy of Viruses (ICTV) first referred to the virus that causes COVID-19 as 2019-nCoV before renaming it as SARS-CoV-2 [2]. The research that is now available implies that COVID-19 has a natural origin and is spread by inhaling the droplets that are produced from the sputum of infected patients. Notably, investigations have suggested that COVID-19 individuals are more likely to have fungus infections. In contrast to earlier reports that included the occurrence of *Candida* bloodstream infection and COVID-19-associated pulmonary aspergillosis (CAPA), a recent finding showed a sharp rise in mucormycosis cases among COVID-19 patients [3-5].

Mucormycosis is caused by fungi called mucormycetes, a dangerous angioinvasive opportunistic infection common in immunocompromised individuals. Rhino-orbital-cerebral, pulmonary, disseminated, cutaneous, and gastrointestinal are the types of diseases included in the spectrum of mucormycosis. Due to climate and the high prevalence of individuals with uncontrolled diabetes, India has the highest number of mucormycosis cases worldwide [6]. Due to the aggressive nature of the infection, mucormycosis detection and therapy is tough; hence, rapid diagnosis, reversal of underlying risk factors (if possible), effective surgical debridement of infected tissue, and suitable antifungal medication are four elements that are essential for curing mucormycosis. Amphotericin B (AmB) is the antifungal of choice for the treatment of mucormycosis due to its effectiveness [7,8].

Diabetes, the foremost common lifestyle disorder, is an independent risk factor for severe COVID-19 and mycosis. Compared to healthy people without chronic conditions, patients with diabetes have greater rates of morbidity and mortality. People who are older and having medical/immunocompromised status including cancer, diabetes, chronic lung disease, or cardiovascular disease are more prone to severe infections that could cause mortality [9]. Hence, this study aims to find the correlation between COVID-19 and diabetes in patients with mucormycosis. The objectives of the study are to analyze the clinical range of mucormycosis in those with diabetes and COVID-19 and to correlate the clinical and radiographic findings.

## Materials and methods

A retrospective, observational, non-interventional, clinical study was carried out at Saveetha Dental College and Hospitals in Chennai (approval number: IHEC/SDC/OMED-2204/23/218). The data collection was done from the institution's electronic database, Dental Information Archiving Software (DIAS), which included a total of 25 samples with a history of mucormycosis. Most commonly, patients with mucormycosis gave a history of swelling and discharge in the affected area. Initial diagnosis was made with cone-beam computed tomography, and final diagnosis was made with histopathological studies with biopsy from the involved site.

The inclusion criteria were patients with mucormycosis, who were 30 years of age or older. Patients under 30, with other fungal infections, were excluded from the study. Database search was conducted using terminologies including mucormycosis, COVID-19, and diabetes. Data were collected from an electronic database from April 2019 to April 2023. All the patients who satisfied the inclusion criteria and with a diagnosis of mucormycosis were included in the study. The data was statistically analyzed using the chi-squared test and Spearman's correlation, and results were obtained.

Statistical analysis

Data was collected in an Excel spreadsheet and analyzed with IBM SPSS Statistics for Windows, Version 23.0 (Released 2015; IBM Corp., Armonk, New York, United States). For quantitative variables, the mean and standard deviation were presented as statistics. The chi-squared tests and Spearman's correlations were used to identify associations between mucormycosis, diabetes, and COVID-19, and a p-value of less than 0.05 is considered to be significant. P-values of 0.01 or lesser are regarded as highly significant. Treatment depends on the severity of the lesion. During the initial stages, AmB and debridement were done, and in severe cases, surgical resection was indicated followed by flap surgery after which patients were followed up for six months. No history of remission was reported among the included cases.

## Results

**Figure 1:**
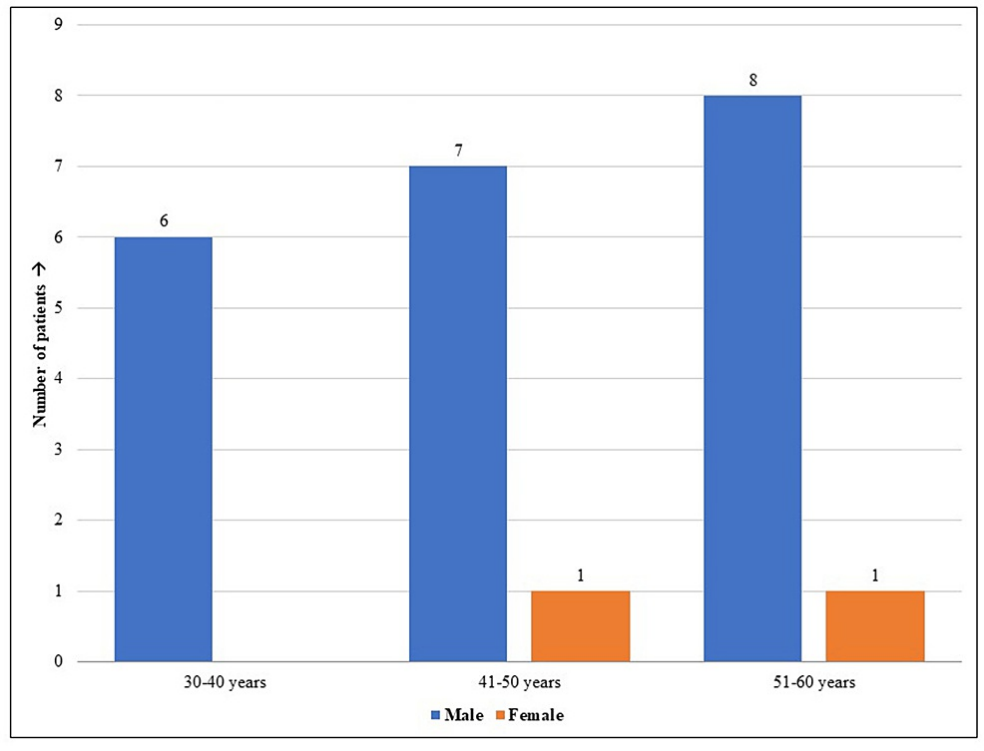
The study population included 22 (88%) male and three (12%) female patients. Among them, 16 (64%) males and all females had both diabetes and mucormycosis.

**Figure 2:**
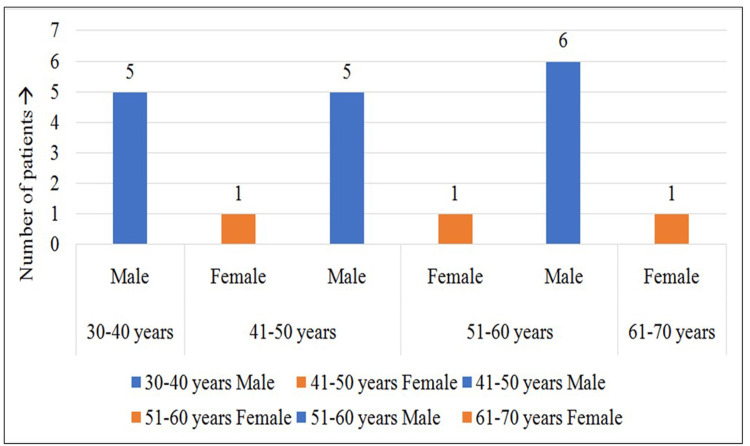
Among the study population, 16 (64%) males and all females had both diabetes and mucormycosis as depicted in the graph.

Twenty-five patients with positive signs of mucormycosis visited the oral medicine department of Saveetha Dental College and Hospitals in Chennai during the study. The patients' average age was 47.76, out of which 22 (88%) were males and three (12%) were females. Among the 25 patients, 19 had diabetes, and 14 had positive COVID-19 history; moreover, 16 (64%) males and all females had both diabetes and mucormycosis. Figure 1 and Figure 2 show the gender distribution and percentage of the study population affected with COVID-19 and diabetes. Table 1 shows that the study population included patients with mucormycosis aged 33-66 years with a mean age of 47.76±10.18 years. One-third of them were aged 51-60 years (36%), followed by 41-50 years (32%) and 30-40 years (28%). 

**Table 1:** The study population included patients with mucormycosis aged 33-66 years with a mean age of 47.76±10.18 years. One-third of them were aged 51-60 years (36%), followed by 41-50 years (32%) and 30-40 years (28%).

	Gender			
	Female		Male	
Age group		n%		n%
30-40 years	0	0	6	24
41-50 years	1	4	7	28
51-60 years	1	4	8	32
61-70 years	1	4	1	4
Total	3	12	22	88

**Figure 3:**
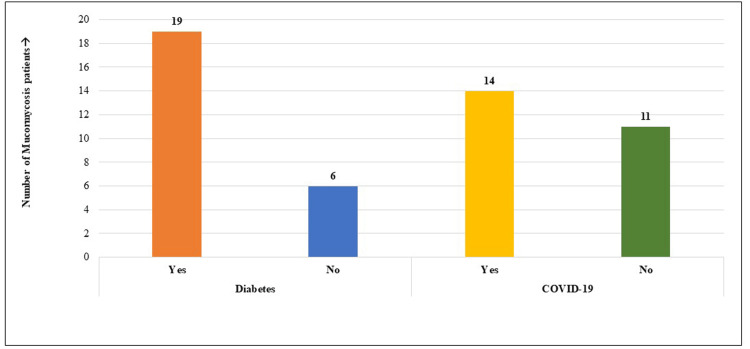
A total of 16 males (64%) and all three females (12%) of the study population had both diabetes and mucormycosis.

**Figure 4:**
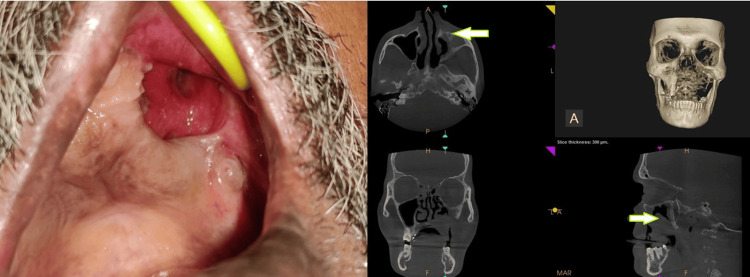
Clinical and radiographic (full skull CBCT) images showing the extent of involvement in mucormycosis. Clinical picture demonstrates the destruction of the maxillary alveolus with the exposure of the underlying tissues. Full skull CBCT image shows osteolytic areas of destruction involving the maxillary alveolus, extending to the maxillary sinus, with the obliteration of the ostiomeatal complex. CBCT: cone-beam computed tomography

**Figure 5:**
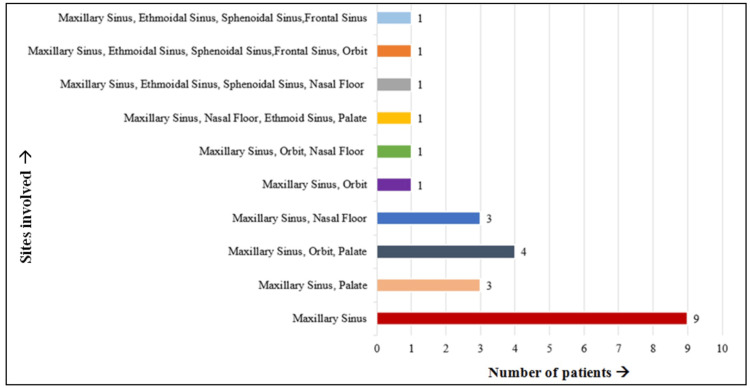
The maxillary sinus is the most common site affected by mucormycosis. All patients had mucormycosis involving the maxillary sinus (100%) along with the palate (32%), orbit (28%), nasal floor (24%), ethmoidal sinus (16%), sphenoidal sinus (12%), and frontal sinus (8%).

A total of 16 males (64%) and all three females (12%) of the study population had both diabetes and mucormycosis (Figure 3). Clinically, patients were reported with headache and pain in relation to the maxillary and nasal region with a history of nasal discharge. Histopathology and cone-beam computed tomography analysis were done to confirm the diagnosis of mucormycosis. Upon evaluation of the radiographic findings, 100% of the patients had involvement of the maxillary sinus, followed by the palate (32%), orbit (28%), nasal floor (24%), ethmoidal sinus (16%), sphenoidal sinus (12%), and frontal sinus (8%), indicating the maxillary sinus as the most common site affected by mucormycosis. Figure 4 and Figure 5 demonstrate the common site of involvement and clinical spectrum of mucormycosis.

Based on age group, it was found that no significant association was found between mucormycosis and diabetes as well as COVID-19 in the present study population (Table 2). The present study also shows no statistically significant relationship between mucormycosis and age (0.285), diabetes (0.234), and COVID-19 (0.643) (Table 3).

**Table 2:** Based on age group, it was found that no significant association was found between mucormycosis and diabetes as well as COVID-19 in the present study population.

		Age group				
		30-40 years n(%)	41-50 years n(%)	51-60 years n(%)	61-70 years n(%)	p-value
Diabetes	Yes	5 (20)	6 (24)	7 (28)	1 (4)	0.816
	No	1 (4)	2 (8)	2 (8)	1 (4)	
COVID-19	Yes	3 (12)	5 (20)	5 (20)	1 (4)	0.968
	No	3 (12)	3 (12)	4 (16)	1 (4)	

**Table 3:** The present study shows no statistically significant relationship between mucormycosis and age (0.285), diabetes (0.234), and COVID-19 (0.643).

		Correlation coefficient (r)	p-value
Age	Diabetes	0.052	0.804
	COVID-19	0.112	0.594
	Mucormycosis	0.222	0.285
Diabetes	Age	0.052	0.804
	COVID-19	0.257	0.216
	Mucormycosis	-0.247	0.234
COVID-19	Diabetes	0.257	0.216
	Age	0.112	0.594
	Mucormycosis	-0.098	0.643
Mucormycosis	Age	0.222	0.285
	Diabetes	-0.247	0.234
	COVID-19	-0.098	0.643

## Discussion

Immunocompromised individuals are the main target of mucormycosis, which is an uncommon yet opportunistic disease. Recent investigations show that older adult males with chronic comorbidities are more susceptible to COVID-19. All three known human pathogenic coronavirus infections, including the severe acute respiratory syndrome coronavirus, are quite severe and are significantly correlated with diabetes [10]. Old age and chronic illnesses like diabetes, hypertension, obesity, coronary artery disease, and heart failure have been linked to worse outcomes in early studies from Asia and Europe [11]. Our study did not show any positive correlation between systemic comorbidities and mucormycosis. According to Patel et al., in India, diabetes makes up 73.5% of all comorbidities with mucormycosis [12].

The COVID-19 pandemic has a significant negative impact on everyone's physical, emotional, social, and economic well-being. Immune imbalance was one underlying factor that connected diabetes to disease severity. According to Roden et al., the radiological pattern of mucormycosis was mainly rhino-orbital or sino-nasal, which was even analyzed, and a similar association was found in the present study [13]. Our study did not align with Hussain et al., showing no bidirectional relationship between diabetes and COVID-19 [14].

Re-infections with COVID-19 following either a natural spread or a vaccination, as seen in Brazil and the United States, respectively, are likely to be caused by new variations [15]. A recent study suggests that the increase in mucormycosis cases could be partially attributed to the heightened use of steroids in COVID-19 patients. The study found that administering dexamethasone to hospitalized COVID-19 patients led to a low mortality rate within 28 days compared to those receiving invasive mechanical ventilation or oxygen alone [16]. The pathogenic mechanisms connected to the development of fungi that are aggressive include the decline in phagocytic function, the increased availability of iron as a result of transferrin's displacement of protons in diabetic ketoacidosis, and the fungal heme oxygenase, which encourages iron intake for its metabolism [17]. Major or mild trauma is thought to pose a serious risk for developing cutaneous mucormycosis, possibly as a result of spore inoculation into the open wound [18]. Recent investigations show that older adult males with chronic comorbidities are more susceptible to COVID-19 [19]. Immune imbalance was one underlying factor that connected diabetes to disease severity [20]. Our study didn't show a bidirectional relationship with diabetes and COVID-19.

Limitations

The study has several limitations, one of which is being conducted at a single center, retrospectively. As a result of the same, we could obtain only a small sample size of 25, and hence, a statistically significant correlation could not be obtained. Moreover, since the study was conducted retrospectively, chances of hyperglycemia secondary to the use of steroids could not be assessed. Further multicenter studies conducted prospectively with long-term follow-up are required for establishing the correlation. Additionally, post-mucormycosis treatment dental considerations should also be taken into account.

## Conclusions

A correlation between mucormycosis, diabetes, and COVID-19 has been already reported in the literature. The findings of this study point out the importance of considering the presence of systemic comorbidities like diabetes in COVID-19 patients. This is important for the identification and treatment of mucormycosis. Early identification, surgical debridement, and antifungal medications are part of the treatment for increased survival.

## References

[1] Mishra Y, Prashar M, Sharma D, Akash Akash, Kumar VP, Tilak TV (2021). Diabetes, COVID 19 and mucormycosis: clinical spectrum and outcome in a tertiary care medical center in western India. Diabetes Metab Syndr.

[2] Jalan S, Gayathri R, Vishnu Priya V, Kavitha S (2020). Awareness on self isolation to prevent COVID 19 infection among elderly people of Tamilnadu - a survey. Int J Curr Res Rev.

[3] Ganesh S, Vishnu Priya V, Gayathri R, Kavitha S (2020). Awareness on dental treatment during COVID-19 among south Indian population. Int J Curr Res Rev.

[4] Divya Sri E, Vishnu Priya V, Hannah R, Gayathri R (2020). Awareness on the role of health care workers in COVID-19 - need of the hour. Int J Curr Res Rev.

[5] Harini M, Devi G, Gayathri R (2020). Awareness among college students towards COVID-19 and its effects on the cardiovascular system - a survey. Int J Curr Res Rev.

[6] Sahu M, Shah M, Mallela VR (2023). COVID-19 associated multisystemic mucormycosis from India: a multicentric retrospective study on clinical profile, predisposing factors, cumulative mortality and factors affecting outcome. Infection.

[7] Jeong W, Keighley C, Wolfe R, Lee WL, Slavin MA, Chen SC, Kong DC (2019). Contemporary management and clinical outcomes of mucormycosis: a systematic review and meta-analysis of case reports. Int J Antimicrob Agents.

[8] Spellberg B, Edwards J Jr, Ibrahim A (2005). Novel perspectives on mucormycosis: pathophysiology, presentation, and management. Clin Microbiol Rev.

[9] Muralidharan VA, Gayatri Devi R, Gheena S (2020). Awareness among college students on the changes in hematological parameters due to COVID-19. Int J Curr Res Rev.

[10] Bornstein SR, Rubino F, Khunti K (2020). Practical recommendations for the management of diabetes in patients with COVID-19. Lancet Diabetes Endocrinol.

[11] Palaiodimos L, Kokkinidis DG, Li W (2020). Severe obesity, increasing age and male sex are independently associated with worse in-hospital outcomes, and higher in-hospital mortality, in a cohort of patients with COVID-19 in the Bronx, New York. Metabolism.

[12] Patel A, Kaur H, Xess I (2020). A multicentre observational study on the epidemiology, risk factors, management and outcomes of mucormycosis in India. Clin Microbiol Infect.

[13] Roden MM, Zaoutis TE, Buchanan WL (2005). Epidemiology and outcome of zygomycosis: a review of 929 reported cases. Clin Infect Dis.

[14] Hussain A, Bhowmik B, do Vale Moreira NC (2020). COVID-19 and diabetes: knowledge in progress. Diabetes Res Clin Pract.

[15] Singh J, Rahman SA, Ehtesham NZ, Hira S, Hasnain SE (2021). SARS-CoV-2 variants of concern are emerging in India. Nat Med.

[16] Dilek A, Ozaras R, Ozkaya S, Sunbul M, Sen EI, Leblebicioglu H (2021). COVID-19-associated mucormycosis: case report and systematic review. Travel Med Infect Dis.

[17] Waizel-Haiat S, Guerrero-Paz JA, Sanchez-Hurtado L, Calleja-Alarcon S, Romero-Gutierrez L (2021). A case of fatal rhino-orbital mucormycosis associated with new onset diabetic ketoacidosis and COVID-19. Cureus.

[18] Jeong W, Keighley C, Wolfe R, Lee WL, Slavin MA, Kong DC, Chen SC (2019). The epidemiology and clinical manifestations of mucormycosis: a systematic review and meta-analysis of case reports. Clin Microbiol Infect.

[19] Huang C, Wang Y, Li X (2020). Clinical features of patients infected with 2019 novel coronavirus in Wuhan, China. Lancet.

[20] Knapp S (2013). Diabetes and infection: is there a link?--a mini-review. Gerontology.

